# Paradigms of endothelial stiffening in cardiovascular disease and vascular aging

**DOI:** 10.3389/fphys.2022.1081119

**Published:** 2023-01-11

**Authors:** Victor M. Aguilar, Amit Paul, Dana Lazarko, Irena Levitan

**Affiliations:** ^1^ Department of Medicine, Division of Pulmonary and Critical Care, College of Medicine, University of Illinois at Chicago, Chicago, IL, United States; ^2^ Richard and Loan Hill Department of Biomedical Engineering, University of Illinois at Chicago, Chicago, IL, United States

**Keywords:** endothelial biomechanics, shear stress, matrix stiffness, oxidized lipids, endothelial barrier

## Abstract

Endothelial cells, the inner lining of the blood vessels, are well-known to play a critical role in vascular function, while endothelial dysfunction due to different cardiovascular risk factors or accumulation of disruptive mechanisms that arise with aging lead to cardiovascular disease. In this review, we focus on endothelial stiffness, a fundamental biomechanical property that reflects cell resistance to deformation. In the first part of the review, we describe the mechanisms that determine endothelial stiffness, including RhoA-dependent contractile response, actin architecture and crosslinking, as well as the contributions of the intermediate filaments, vimentin and lamin. Then, we review the factors that induce endothelial stiffening, with the emphasis on mechanical signals, such as fluid shear stress, stretch and stiffness of the extracellular matrix, which are well-known to control endothelial biomechanics. We also describe in detail the contribution of lipid factors, particularly oxidized lipids, that were also shown to be crucial in regulation of endothelial stiffness. Furthermore, we discuss the relative contributions of these two mechanisms of endothelial stiffening in vasculature in cardiovascular disease and aging. Finally, we present the current state of knowledge about the role of endothelial stiffening in the disruption of endothelial cell-cell junctions that are responsible for the maintenance of the endothelial barrier.

## 1 Introduction

Deterioration of vascular function with age or vascular aging is recognized today as a major factor in the aging process, whereas maintaining vascular health is considered a promising direction in developing anti-ageing strategies (Perk et al., 2012; Kajuluri et al., 2021). A major characteristic of vascular aging and an independent predictor of cardiovascular disease and mortality is stiffening of large elastic arteries, particularly aorta, which increases throughout the normal life span and is exacerbated by cardiovascular risk factors, such as obesity and diabetes (Laurent et al., 2001; Mitchell et al., 2007; Mitchell, 2008; Palombo and Kozakova, 2016). Aortic stiffening is also associated with age-related cognitive decline (Waldstein et al., 2008; Tarumi et al., 2013), as well as with kidney damage (Safar et al., 2004). A major link between the stiffening of large arteries and functional vascular dysfunction is the impairment of peripheral microvascular function, which is manifested in decreased flow-induced vasodilation and has been demonstrated in several large cohorts of human subjects including the Framingham study (Mitchell et al., 2005; Cheung et al., 2007). Arterial stiffening results in reduced compliance of the arterial vascular wall to changes in blood pressure and blood velocity and thus, creates abnormal shear stress patterns that might be damaging to the endothelium (Mitchell, 2008). The stiffening of the arterial wall may also lead to increased contractility of the endothelium, which in turn impairs the integrity of the endothelial monolayer (Kohn et al., 2015a)**.**

Arterial stiffness is primarily determined by the aggregate stiffness of the multi-layers of vascular smooth muscle cells (VSMCs), the major cellular component of the arterial wall, and by the composition of extracellular matrix. Typically, extracellular stiffening is a result of the loss and fragmentation of elastin and depositions and crosslinking of collagen by advanced glycation end-products (AGEs) (Kohn et al., 2015a; Palombo and Kozakova, 2016), and VCMCs stiffening is a result of the cytoskeletal remodeling (Vatner et al., 2021). Importantly, endothelial stiffness is critically distinct from a more thoroughly studied stiffness of the vascular wall. Mechanically, the stiffness of the endothelium, a single cell monolayer that constitutes the inner lining of the blood vessels is not expected to contribute to the general stiffness of the vascular wall. However, an increasing amount of evidence suggests that endothelial stiffness *per se* has significant physiological impact on the vascular function, including disruption of the endothelial barrier and recruitment of monocytes to the sites of inflammation, two major endothelial functions. There are also multiple connection between VCMCs and ECs which contribute to regulating arterial stiffening: on one hand, an increase in VCMC stiffness contribute to the stiffening of the arterial wall, which in turn induce endothelial stiffening, and on the other hand, endothelial stiffening may impair endothelial vasodilatory function, which would enhance the contractility of VCMCs contributing to vascular stiffness. It is critical, therefore, to understand the mechanisms that govern endothelial stiffness and determine the contribution of endothelial stiffening to vascular aging. In this review, we discuss the current state of knowledge about the factors and the mechanisms that govern endothelial stiffness in general and in aging vasculature.

## 2 Determinants of endothelial cortical stiffness

Cellular stiffness is a fundamental biomechanical property that reflects the elasticity and deformability of the cellular envelope, a bi-component system consisting of the membrane lipid bilayer and the sub-membrane cytoskeleton, also known as the cytoskeletal cortex. It is well known that membrane lipid bilayer is a much softer material than the cytoskeleton and therefore it is the sub-membrane cortex that constitutes the cellular mechanical scaffold. Hence, cellular stiffness is frequently referred to as cortical stiffness. The three major cytoskeletal networks in mammalian cells are microfilaments (∼7 nm diameter) comprised primarily of actin filaments, microtubule (∼25 nm diameter) comprised of tubulin and intermediate filaments; (∼10 nm diameter), which can be comprised of several types of proteins with vimentin being the major one in endothelial cells (Berhadsky and Vasiliev, 1989). The biomechanical properties of the three networks were shown to be clearly distinct, as assessed in isolated filaments: the greatest resistance to stress is exhibited by actin filaments that have highest rigidity but fluidize at high strains, vimentin filaments are less rigid but stiffen at higher strains and microtubule, while relatively stiff were predicted not contribute significantly to cellular viscoelastic properties because of their low abundance in cellular cortex (Janmey et al., 1991).

### 2.1 Actin cytoskeleton

Actin cytoskeleton is a network of actin filaments, myosin and different actin-binding proteins, which constitutes the main component of the sub-membrane cytoskeleton or the cell cortex, and exists under continuous tension that arises from the activity of myosin motors (Morone et al., 2006). Multiple studies established that endothelial deformability also depends primarily on actin cytoskeleton. First, using micropipette aspiration, which measures membrane/cortical deformation in response to negative pressure applied to the cell surface, it was demonstrated that disruption of F-actin either with cytochalasin B, a capping protein, that interferes with the formation of actin filaments or with latrunculin A, which binds to G-actin, result in significant endothelial softening, disruption of the tubule has little or no effect (Sato et al., 1990; Byfield et al., 2004). Substrate adherent capillary endothelial cells were also shown to be under internal mechanical tension, as they spontaneously retracted when cut across by a microneedle (Pourati et al., 1998). This pre-existing tension was largely abrogated by disrupting the integrity of F-actin, indicating that actin is the stress bearing element of the cytoskeleton. Furthermore, actin tension was also shown to correlate with cell stiffness, as determined by magnetic twisting cytometry, and proposed to constitute a key determinant of cellular deformability (Pourati et al., 1998). Furthermore, disruption of F-actin also significantly decreases the force required to pull membrane tethers in aortic endothelial cells indicating that it is a major determinant of membrane biomechanics and membrane-cytoskeletal adhesion (Sun et al., 2007). Two general mechanisms are known to contribute to increased cortical tension/cortical stiffness: 1) increase in actomyosin contractility and stress fiber formation, 2) increase in the abundance and/or the crosslinking of cortical actin.

Actomyosin-mediated contractility is a major mechanism for generating mechanical stress and increased cortical tension in all mammalian cells (Heissler and Manstein, 2013; Murrell et al., 2015). In my cells, the contractility occurs as a result of the interaction between myosin II molecular motor and actin filaments and is determined by the phosphorylation status of myosin light chain (MLC) regulatory domain of myosin II (Heissler and Manstein, 2013; Murrell et al., 2015). MLC phosphorylation promotes myosin II activity and triggers the assembly of stress fibers, actomyosin bundles that have contractile properties. The upstream regulators of MLC phosphorylation are RhoA/Rho-kinase coiled-coil containing kinase (ROCK) pathway and myosin light chain kinase (MLCK), which in turn can be activated by a variety of factors.

Numerous studies showed that activation of RhoA/ROCK pathway induces formation of stress fibers and enhances cellular contractility [e.g., (Ridley and Hall, 1992; Totsukawa et al., 2000)]. The major pathway by which ROCK phosphorylated MLC is not direct but mediated *via* the inhibition protein phosphatase 1 (PP1 or MLCP), which dephosphorylates (Matsumura and Hartshorne, 2008). MLCK activation is Ca/Calmodulin dependent and directly phosphorylates MLC (Takashima, 2009). Initially, it was shown through indirect intracellular cytoplasmic microrheology measurements that an increase in the nanoscale intracellular viscosity of fibrobasts was correlated with an increase in RhoA activity (Kole et al., 2004). In a later study, it was directly demonstrated that activation of RhoA/ROCK increases stress fiber formation, cytoskeletal tension, and cell stiffness, as thoroughly assessed by Lim et al. (2012). More specifically, Atomic Force Microscopy (AFM) was utilized with AFM probe tips coated with ECM (laminin or fibronectin) so that contact with vascular smooth muscle cells (VSMCs) would result in cell adhesion *via* focal adhesion (FA) formation. Cells were mechanically stimulated by systematically retracting the AFM probe attached to the FA. By utilizing this AFM technique in conjunction with VSMCs transfected to fluorescently express vinculin, RhoA, and actin, the authors were able to elegantly demonstrate that direct mechanical stimulation led to an increase in localized RhoA activity at the site of stimulation, which subsequently led to increases in F-actin stress fiber activity and cellular tension. They utilized loss of function/gain of function of RhoA constructs for the RhoA-EGFP transfections: a dominant negative version (low RhoA), and a constitutively active version (high RhoA) and a wild type as control. Cells with the dominant negative version had very low cell stiffness compared to the wild type variety, and cells with the constitutively active version had higher stiffness values. With robust retraction of the AFM probe (simulating a high application of tensile stress), the dominant negative RhoA cells detached from the AFM probe very quickly after the initial adhesion. Meanwhile, the constitutively active RhoA cells were found to strongly resist the high tensile stress application of the AFM probe retraction. They also demonstrated that blocking Rho-kinase *via* the specific inhibitor Y-27632 or blocking myosin function by the myosin inhibitor ML-7 led to low cytoskeletal tension with decreased levels of stress fibers, cytoskeletal tension, and cell stiffness. In more recent studies, CN03 has been utilized as a potent Rho activator, which constitutively activates Rho by blocking GTPase activity without affecting the activity or Rac and Cdc42 (Bade et al., 2017). Showed that CN03 greatly enhances basal stress fiber formation, which has been previously reported to be the primary sub-category of stress fibers associated with cell tension and stiffness (Ingber and Tensegrity, 2003; Kumar et al., 2006). Interestingly, the intracellular spatial distribution of RhoA/ROCK and MLCK was shown to be distinct resulting in differential patterns of stress fiber formation: the assembly of the stress fibers in the center of the cells was shown to be mainly ROCK dependent, whereas in the periphery, it was shown to be dependent more strongly on MLCK (Totsukawa et al., 2000). This raises a possibility that different stimuli that activate these pathways are also likely to generate spatially distinct stiffening patterns.

### 2.2 Cortical actin architecture

While many studies assume that cortical tension is determined primarily by the expression and phosphorylation levels of myosin with actin fibers only providing the scaffold for myosin activity (Tinevez et al., 2009), growing number of studies indicate that actin architecture also plays a major role in determining the cortical tension. Specifically, it was shown that altering actin architecture using surface micropatterning and the network organization of the fibers play major roles in the contractile response and that specific actin crosslinkers (also known as actin binding proteins) can enhance or inhibit the contractility under the same expression/phosphorylation levels of myosin (Reymann et al., 2012; Ennomani et al., 2016). Additionally actin binding proteins have been previously well-established to mediate F-actin stiffness at the cell’s leading edge cortical actin bundles (Dos Remedios et al., 2003; Uribe and Jay, 2009; Pollard, 2016; Vakhrusheva et al., 2022). In one particular study, α-actinin, plastin, or fascin were varied for their concentration and bundle size (these specific actin binding proteins are abundant in eukaryotic cells) (Claessens et al., 2006). Leading edge cortical actin bundle stiffness was precisely measured and found to differ uniquely and significantly depending on the specific type of actin binding protein being manipulated. In addition, the thickness of the cortical actin was also shown to be a determinant of cortical tension independently of myosin and most interestingly, the relationship between cortical thickness and tension was found to be non-monotonic with maximum tension generated at medium cortical stiffness (Chugh et al., 2017). It is expected, therefore, that changes in the expression levels of multiple actin binding proteins that regulate actin architecture have significant impacts on cortical tension and further systematic studies are needed to elucidate the roles of different actin binding proteins in cortical contractility and stiffness.

### 2.3 Vimentin intermediate filaments

In addition to actin filaments, vimentin filaments were also shown to contribute to the viscoelastic properties of several cell types, including endothelial cells but its contribution is more subtle (Wang and Stamenovic, 2000; Mendez et al., 2014). Using magnetic twisting cytometry, it was shown that disrupting intermediate filaments with acrylamide results in softening in capillary endothelial cells, even though the effect appears to be milder than that of F-actin disruption (Wang and Stamenovic, 2000). A similar effect was also observed in vimentin-deficient fibroblasts (Wang and Stamenovic, 2000; Mendez et al., 2014). The loss of vimentin was also shown to reduce cell elasticity and increase heterogeneity of the viscoelastic properties, which led to a hypothesis that vimentin has a protective role in maintaining cellular biomechanical phenotype, particularly when exposed to mechanical stresses (Mendez et al., 2014). Most recently, wild-type and vimentin-null mouse embryonic fibroblasts were utilized to study the protective effects of vimentin on the nucleus. Notably, it was demonstrated that the loss of vimentin causes significant nuclear deformations, and vimentin ^−/−^ cells were approximately 20% softer in the perinuclear region than the wild-type counterparts (Patteson et al., 2019). Rescue with vimentin overexpression of the vimentin ^−/−^ cells restored nuclear shape and perinuclear stiffness, further solidifying the protective role of vimentin and it is contribution to cell stiffness. Additionally, oncogenes have been shown to alter the spatial organization of vimentin, and changes in oncogene expression lead to specific changes in vimentin configuration (without altering other cytoskeletal components), which in turn leads to changes in cell stiffness (Rathje et al., 2014).

Lamins are a group of type V intermediate filaments, which have been a focus of cell stiffness related studies for the past decade. Briefly, the nucleoskeletal protein lamin a/c increases in a scaled manner with matrix/tissue stiffness and contributes to nuclear stiffness and cell stiffness at the perinuclear region (Swift et al., 2013; Srivastava et al., 2021). Although a few rudimentary studies have been performed to assess cell stiffness when microtubules are disrupted (Hobson et al., 2020), the role of microtubules in cell stiffness remains relatively unexplored.

**FIGURE 1:**
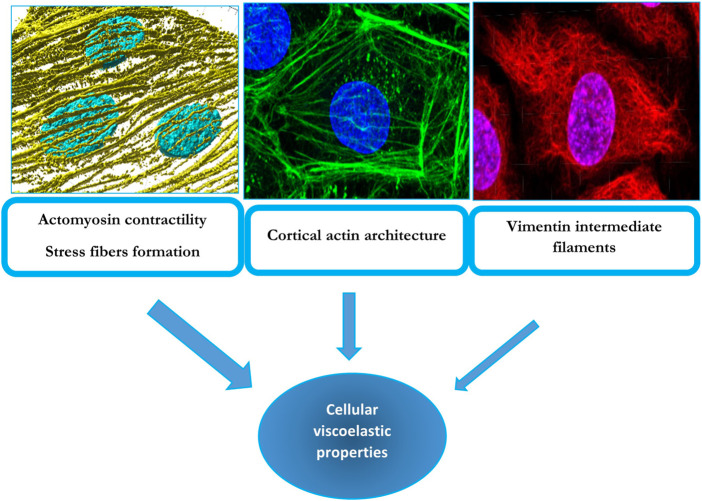
Cytoskeletal determinants of cellular viscoelastic properties: F-actin stress fibers (left top panel), cortical actin (middle top panel) and vimentin intermediate filaments (right top panel). The top left panel displays a 3D render of F-actin stress fibers (yellow) and nuclei (blue) from endothelial cells undergoing alignment following exposure to laminar flow. These fibers also contain non-muscle myosin and underlie cellular contractility, which has a major contribution to endothelial biomechanics. The middle panel shows an endothelial cell with a strong cortical/junctional F-actin network (green) and nuclei (blue), which was also found to contribute to passive biomechanical properties of endothelial cells. The right panel shows an endothelial cell’s nuclei (blue), and vimentin intermediate filament network (red) which has a smaller but still significant contribution to endothelial biomechanics. The width of the arrows conveys the degree of the contribution of the particular cytoskeleton element to endothelial viscoelastic properties. The contributions of these elements to endothelial stiffness are described in Sections 2 and Section 6 of the manuscript.

In conclusion, the current state of knowledge is that endothelial stiffness depends primarily on actomyosin-mediated contractility with emerging roles of actin architecture and crosslinking and possibly of vimentin and lamin intermediate filaments (Figure 1).

## 3 Endothelial stiffening in response to mechanical signals

**FIGURE 2:**
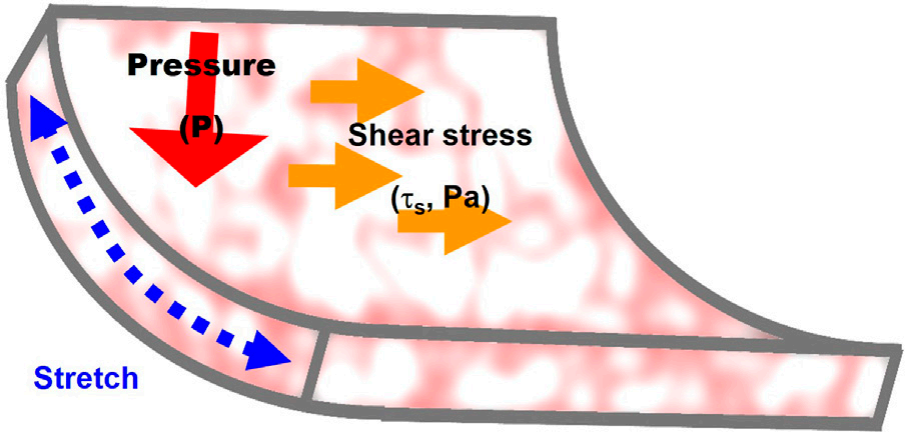
Mechanical forces that regulate endothelial stiffening: 1) stretch generated by contraction/relaxation of the arterial wall, 2) fluid shear stress, a frictional force generated by the blood flow, and 3) hydrostatic pressure. The mechanisms involved in the effects of the three forces on endothelial stiffness are described in Section 3 of the manuscript.

Endothelial lining of the blood vessels is continuously exposed to mechanical loads: fluid shear stress, a frictional force generated by the blood flow, hydrostatic pressure generated by blood pressure, and circumferential cyclic stretch during constriction/relaxation cycles of the arteries (Figure 2). Endothelium also experiences significant changes in the sub-endothelial stiffness, as a result of vascular remodeling in different disease state and during aging.

### 3.1 Fluid shear stress

Shear stress is well known to be one of the primary factors in endothelial function with laminar unidirectional flow being anti-inflammatory and disturbed non-unidirectional flow being pro-inflammatory (Davies et al., 2005; Grady et al., 2016). It is also well-known that unidirectional flow is atheroprotective while the non-unidirectional flow predisposes the arterial regions to atherosclerosis development. Noteworthy, disturbed flow is also an aging factor and induces premature aging (Hahn and Schwartz, 2009).

### 3.2 Laminar unidirectional flow

Numerous studies have shown that exposure of endothelial cells to laminar flow results in the formation of thick actin stress aligned in the direction of flow (Grady et al., 2016; Dominic et al., 2020). A transition from the static culture to laminar flow was also shown to induce endothelial stiffening, as estimated first by micropipette aspiration (Sato et al., 1987; Davies, 1995) and then by the indentation-based Atomic Force Microscopy (Sato et al., 1996; Sato et al., 2000). Interestingly, upon the transition from static to laminar shear stress conditions, the stiffening response develops first in the part of the front edge of the cell that faces the flow in non-monolayer cell cultures and then later equilibrates to the rest of the cell (Sato et al., 1996). Mechanistically, laminar shear stress is known to activate RhoA GTPases, specifically, RhoA and Rac (Tzima et al., 2001; Ohashi et al., 2002; Tzima et al., 2002), both key regulators of actin cytoskeleton dynamics and architecture (Hall 1998; Wojciak-Stothard and Ridley, 2003; Hall, 2012; Ridley, 2015). As described above, activation of RhoA induces formation of actin stress fibers and activation of endothelial contractile response, whereas activation of Rac promotes formation of cortical actin and lamellipodia growth (Wojciak-Stothard and Ridley, 2003; Hall, 2012; Ridley, 2015). Activation of RhoA by laminar shear stress was shown to be biphasic, with a transient decrease of RhoA activity at the initiation of the flow, followed by an enhanced activity (Tzima et al., 2002). This biphasic response, coupled with the activation of Rac was proposed to be essential for flow-induced endothelial alignment: a transient decrease in Rho activity inhibits myosin-dependent contractility and disassembly of the stress fibers, allowing endothelial cells to change shape and grow lamellipodia in the direction of the flow, which is mediated by Rac at the edge of the cells downstream of the flow; then, the delayed phase of Rho activation strengthens and stabilizes the new cell shape and alignment by the formation of shear stress fibers in the direction of the flow (Civelekoglu-Scholey et al., 2005).

### 3.3 Disturbed non-unidirectional flow

Physiologically, however, a more relevant question is not what happens when cells maintained under static conditions are exposed to flow but whether laminar and disturbed flows have distinct effects on endothelial stiffness. Again, it is very well-known that the architecture of the cytoskeleton is vastly different in cells exposed to unidirectional/laminar and non-unidirectional/disturbed flow: while as described above, laminar flow induces formation actin stress fibers aligned in the direction of flow, exposure to disturbed flow results in abundant stress fibers without clear orientation or direction. In terms of endothelial stiffness, however, studies from our lab showed no difference in the elastic properties of endothelial cells, as measured by the AFM, when cells were exposed to the two types of flow, unless the cells were also exposed to oxidized low-density lipoproteins (oxLDL) (LeMaster et al., 2018). The mechanisms and the physiological implications of oxLDL-induced endothelial stiffening and its dependence on the hemodynamic environment are described in detail later in this review.

Stretch was also shown in earlier studies to induce endothelial stiffening, which was attributed to an increase in cytoskeleton tension (Pourati et al., 1998). It was also shown that cyclic biaxial mechanical stretch results remodeling of adherens junctions from linear to zipper-like, which is a known characteristic of junction under tension and in the up-regulation of myosin light chain-2 (pMLC2) phosphorylation, which as described above is indicative of increased actomyosin contractility, and increase in cell stiffness (Miroshnikova et al., 2021). Furthermore, it was shown that the process is biphasic, with the initial contractile response driven by the influx of Ca^2+^ *via* stretch-sensitive Piezo1 channels leading to the activation of Rho-GTPases, and the second phase driven by phosphorylation of actin cross-linker proteins filamins (Miroshnikova et al., 2021).

Hydrostatic pressure was also shown to induce an increase in endothelial stiffness. In an *in vitro* model of short-term (24 h) high pressure exposure, aortic ECs underwent significant structural changes, specifically formation of stress fibers, as identified by phalloidin staining suggesting an increase in cellular stiffness (Ohashi et al., 2007). Computational modeling of stress fiber networks in pulmonary microvascular ECs suggested that stress fiber stiffness (specifically bending stiffness) contributes to the generation of pressure-induced cellular high strain levels (Ito et al., 2010). Furthermore, inducing strain in these cells in culture using a uni-axial stretching apparatus, which was predicted to increase cell strain, resulted in stress fiber formation, which was dependent on the activation of stretch-activated cation channels (Ito et al., 2010). More recently, significant endothelial stiffening was observed in response to increased hydrostatic pressure generated by a custom-made small volume pressurized chamber that allows exposing live cells to controlled pressure (Prystopiuk et al., 2018). The stiffening was observed in response to both acute (1 h) and chronic (24 h) hydrostatic pressures of 100 mm Hg (13 kPa), with stiffening still observed 1 hour post recovery in cells exposed to chronic high pressure exposure. The stiffening response was accompanied by the increase of retraction velocity of cortical filaments and abrogated by inhibiting myosin II phosphorylation with blebbistatin, both indicating that it is mediated by myosin II-dependent contraction. The high pressure-induced endothelial stiffening was also abrogated by blocking mechanosensitive cation channels and blocking or downregulation ENaC sodium channels. The authors conclude that hydrostatic pressure induced endothelial contractile response *via* Ca^2+^ influx through mechanosensitive ion channels and Ca^2+^-mediated activation of myosin.

There are also studies that suggest that generation of intracellular pressure may result in cell stiffening. Beyder and Sachs (2009) showed that the application of hydrostatic pressure through a micropipette directly to the cell interior in a whole-cell patch configuration results in a decrease in membrane deformability, which indicates membrane stiffening. Consistent with this study, we found that hypotonic challenge results in endothelial stiffening, which was not abolished but rather enhanced by the depolymerization of actin cytoskeleton, which led us to hypothesize that the stiffening is a result of an increased hydrostatic pressure that develops from osmotic influx into the cells (Ayee et al., 2018).

### 3.4 Extracellular matrix stiffness

Extracellular matrix is a highly heterogenous milieu of interconnecting fibers that provides mechanical support for all tissues. It is well established that cells sense the stiffness of the ECM and respond to it in multiple ways that include changes in cellular biomechanical properties. In what is considered an adaptive response to the ECM stiffening, cell undergo changes in the cytoskeletal structure to reinforce the internal scaffold by increase in stress fiber formation and activation of myosin-mediated contractility, which in turn increases cellular cortical stiffness (Wang et al., 2020; Yi et al., 2022). The balance between the ECM stiffness and the cytoskeletal stiffness/tension is believed to be required to maintain cell shape and adhesion to the substrate. The stiffness of the matrix was also shown to determine stem cell lineage to different fates with cell stiffnesses of the differentiated cells to match the elasticity of the substrate (Engler et al., 2006). Mechanistically, stiff substrates lead to increased binding of integrins to the extracellular matrix, increased expression of the component of the focal adhesion, and activation of focal adhesion kinase (FAK), which in turn activates RhoA/myosin-induced contractility (Discher et al., 2005; Yeung et al., 2005). Thus, stiffer substrates generate increased cytoskeleton tension, which manifests itself in increased cellular cortical stiffness.

Our earlier studies demonstrated that substrate stiffness also leads to increased stiffness of endothelial cells, both in 2D and in 3D environments (Byfield et al., 2009). To test the impact of substrate stiffness in a 2D culture, endothelial cells are grown on polyacrylamide gels with elastic moduli between ∼1.5 and 10 kPa, a typical range of stiffness in arteries (Woodrum et al., 2006; Le Master et al., 2018), which led to ∼2 fold increase in endothelial cells, as estimated by micropipette aspiration technique (Byfield et al., 2009). A similar stiffening effect was also observed when endothelial cells were embedded in 3D collagen gels to mimic migration of cells into a tissue, which occurs during formation of new vessels. It was subsequently shows that growing endothelial cells on stiffer substrates also increases endothelial force generation, as assessed by force traction microscopy, an effect that was abrogated by the inhibition or downregulation of Rho-associated kinase, ROCK (Huynh et al., 2011; Krishnan et al., 2011). Also, more recently and consistent with the previous studies, it was shown that on soft anisotropic scaffolds endothelial cells form actin filaments aligning with the direction of the substrate fibers and organized into cortical bands, while on stiffer scaffolds, the cells formed stress fibers spanning the entire cell, which is typical for higher contractile state (Yi et al., 2020). Thus, it is abundantly clear that endothelial stiffness and contractility increase when cells are exposed to stiffer extracellular milieu.

### 3.5 Cell stiffness may modulate the mechanical properties of the extracellular matrix

It is also important to note that the crosstalk between substrate and cellular stiffness is bidirectional: not only increased substrate stiffness provides mechanical cues for cells to stiffen but also cellular traction forces induce remodeling of the matrix. Specifically, it was shown that MLC-dependent cell traction forces are involved in the remodeling of collagen by binding the fibers to protruding lamellipodia and then retracting (Meshel et al., 2005). RhoA/ROCK activation was also shown to induce the assembly of fibronectin fibers by tension-induced exposure of cryptic sites (Zhong et al., 1998) and also by the cytoskeletal tension that is transmitted to the extracellular environment *via* integrins (Singh et al., 2010). Furthermore, an increased rigidity of the substrate promotes the formation of fibrillar fibrinogen matrix (Carraher and Schwarzbauer, 2013), consistent with the increased activation of RhoA and increased cortical tension on stiffer substrates described above. Formation of a mature detergent-insoluble fibrinogen matrix contributes to tissue stability and rigidity, as well as serves as a scaffold for the deposition of other components of extracellular matrix including collagen, which further increase the rigidity/stiffness of the tissue (Singh et al., 2010). Together, these mechanisms provide a bidirectional regulation of tissue/cellular stiffness.

## 4 Endothelial stiffening in response to oxidized lipids

Studies of our group focused over the last decade on the impact of cholesterol and oxidized lipids on endothelial stiffness and contractility. In an early study by Byfield et al. (2004), we established that in contrast to a lipid bilayer that stiffens as a result of the increase in membrane cholesterol, endothelial cells became less deformable/more stiff when cellular cholesterol was depleted using a cyclic oligosaccharide, methyl-β-cyclodextrin (MβCD) with high-affinity to cholesterol. The stiffening effect of MβCD was determined by micropipette aspiration, an effect that can be reversed upon repletion with membrane cholesterol. The stiffening effect induced by cholesterol depletion was fully abrogated by disruption of F-actin with latrunculin-A (a macrolide which prevents actin polymerization), suggesting that depleting cholesterol increases cell stiffness by altering F-actin and its structural relationship with the cell membrane. Indeed, in a later study, we found that cholesterol depletion significantly increases the adhesion energy between the membrane and the underlying cytoskeleton, while cholesterol enrichment had the opposite effect, as was determined by pulling membrane nanotubes using AFM (Sun et al., 2007). This observation was also unexpected because depleting membrane cholesterol is well-known to disrupt the signaling platforms termed lipid rafts, which are the focal points of membrane-cytoskeletal interactions (Levitan and Gooch, 2007). Consistent with these findings, cholesterol depletion was also found to result in an increase in cytoskeletal traction forces and focal adhesions (Norman et al., 2010). These paradoxical relationships between the effects of cholesterol on stiffness of the lipid bilayer and on cellular biomechanics, which is dominated by the cytoskeleton, are discussed in details in our earlier review (Ayee and Levitan, 2016). The physiological significance, however, of endothelial stiffening induced by cholesterol depletion is not really clear.

**FIGURE 3:**
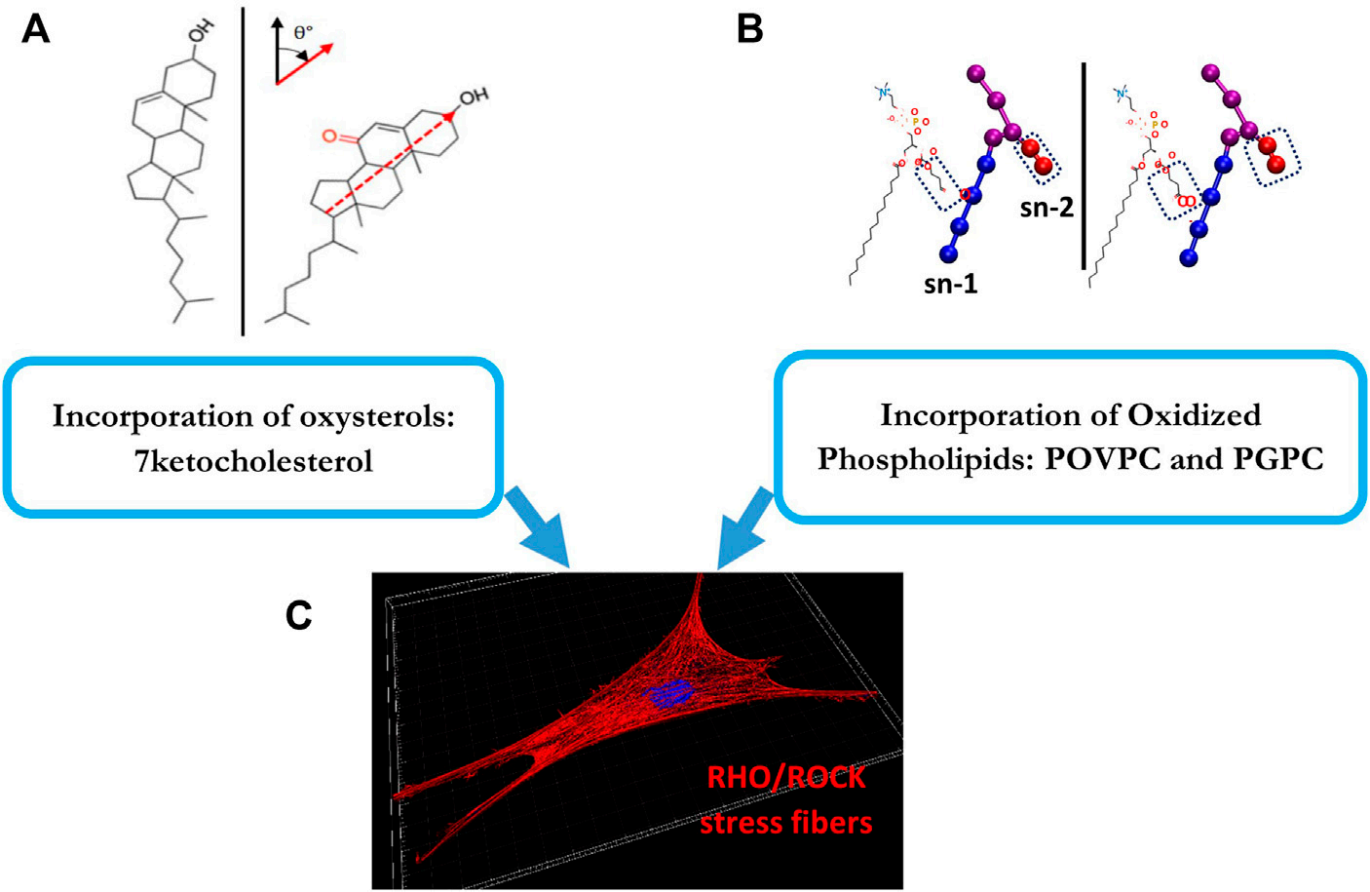
Oxidized lipids that induce endothelial stiffening: The top panels **(A,B)** show the structures of the major oxidized lipids that contribute to endothelial stiffening: a 7-ketocholesterol (left) and oxidized phospholipids, POVPC and PGPC (right). **(A)** shows the structures of cholesterol (left) and 7-ketocholesterol, as evident from the additional carbonyl group, (right) shown at their orientation/tilt in the lipid membrane [adopted from Ayee et al., 2021 as published in *Front Cardiovasc Med* 8 (Ayee and Levitan, 2021)]. **(B)** shows the chemical structures and coarse-grain topologies of oxidized lipid molecules POVPC (left) and PGPC (right) with polar headgroups and glycerol backbones shown as purple spheres, sn-1 tails as blue spheres and sn-2 truncated oxidized tails (circled) as red spheres [adopted from Ayee et al., 2017 as published in *Biophys J* 112 (2) (Ayee et al., 2017b)]. **(C)** shows endothelial stress fibers that are induced as a result of the incorporation of both types of these oxidized lipids into the plasma membrane *via* activation of RhoA/ROCK cascade. This is described in Section 4 of the manuscript.

We turned, therefore, to study the effects of oxidized lipids, such as oxidated modifications of low-density lipoproteins (oxLDL), whose major impact on endothelial function and cardiovascular disease is well established (Levitan et al., 2010). In terms of the relationship between oxLDL and cellular cholesterol, while it has been generally believed that oxLDL loads cells with cholesterol, this notion is controversial, as several studies, found that exposure to oxLDL actually does not load endothelial cells with cholesterol, but instead results in significant cellular increase in oxysterols (Byfield et al., 2006; Shentu et al., 2010; Ayee and Levitan, 2021). Furthermore, this effect is not unique to endothelial cells, as a similar phenomenon was recently reported in macrophages (Rezende et al., 2022). There are also interesting similarities between the effects of oxLDL and exposure and MβCD: in both cases, in addition to causing endothelial stiffening, both oxLDL and MβCD resulted in the internalization of a lipid raft marker GM1 protein, an increase in endothelial force generation and network formation (Byfield et al., 2006), as well as facilitates the alignment of endothelial cells to flow (Kowalsky et al., 2008). These observations indicated that exposure to oxLDL leads to functional/biomechanical effects similar to cholesterol depletion rather than cholesterol enrichment. To further investigate the biophysical basis of this phenomenon, we assessed the degree of lipid order of the plasma membrane using Laurdan imaging, a dye that is sensitive to the presence of water dipoles in the membrane and thus is used to assess lipid order (Levitan and Shentu, 2011; Levitan, 2021). We found that similarly to cholesterol depletion, exposing cells to oxLDL resulted in the disruption of the lipid order, as indicated by a significant shift in the lipid order of the membrane lipid bilayer to a less ordered state (Shentu et al., 2010). In terms of specific lipid species that cause lipid disorder and endothelial stiffening, we found that these effects can be mediated either by oxysterols of by oxidized phospholipids and are a result of the tilt of the specific lipid molecules within the lipid bilayer (Shentu et al., 2012; Ayee et al., 2017a; Ayee and Levitan, 2021). It is also important to note that subtle increases in the amount of oxysterols that are consistent with the elevation of these species under dyslipidemic conditions are sufficient to cause significant molecular-scale biophysical perturbations of the membrane structure resulting in increased cellular stiffness (Ayee and Levitan, 2021). We also established that oxidized lipids induce endothelial stiffening by activating the RhoA/ROCK pathway and formation of stress fibers (Oh et al., 2016), which is well-known to induce cell stiffening, as described above (Figure 3). Noteworthy, an increased uptake of oxLDL under disturbed flow conditions leads to differential stiffening of endothelial cells exposed to laminar and disturbed flow and is also responsible for endothelial stiffening in the aortic arch *in vivo* (LeMaster et al., 2018).

## 5 Endothelial stiffening in aging and cardiovascular disease

**FIGURE 4:**
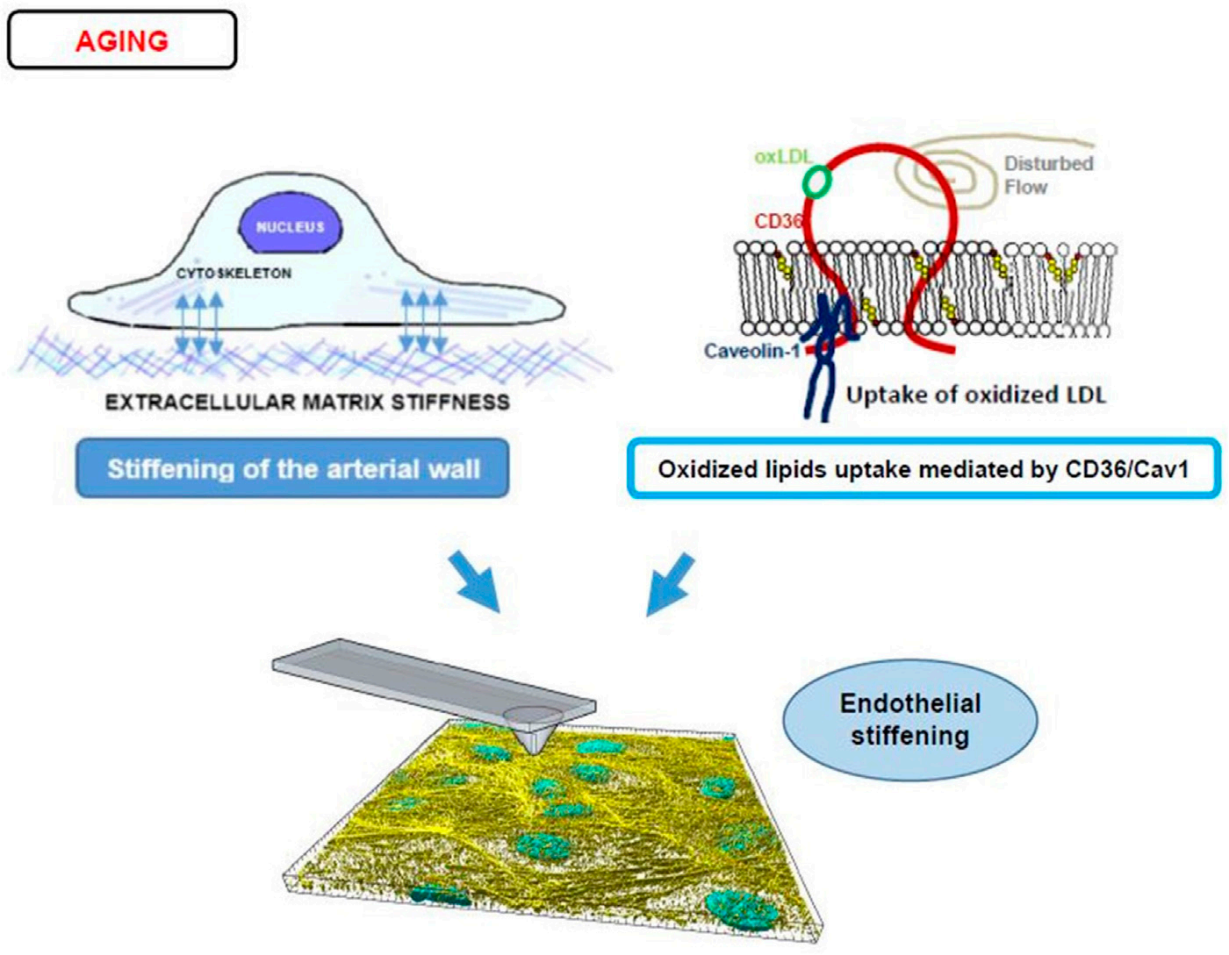
Endothelial stiffening and aging. The top left panel displays a diagram of an endothelial cell’s mechanotransduction process from the ECM (depicted as short crosslines outside of the basal side of a cell) to the focal adhesions/integrins (blue double-sided arrows) to cytoskeletal networks (intracellular light purple bundles). For a stiffer arterial wall and therefore stiffer ECM, this mechanotransduction leads to more F-actin stress fiber activity and stiffer cells. The right top panel displays an alternate process of stiffening caused by disturbed flow and CD36/Cav1 mediated uptake of oxLDL (CD36 is shown schematically as a red integral protein, that is bound to Caveolin-1 at the inner leaflet of the membrane, shown in blue, oxLDL bound to CD36 is shown in green and oxysterols incorporated into the bilayer are shown in yellow/red). The bottom panel shows a 3D reconstructed image of the F-actin stress fibers (yellow) and nuclei (blue) in an endothelial monolayer being probed by an AFM tip to obtain Young’s modulus values. The interplay between ECM and oxLDL in the induction of endothelial stiffening is described in Section 5 of the manuscript.

While numerous studies demonstrated that aging and development of the cardiovascular disease (CVD), such as hypertension and atherosclerosis, are accompanied with arterial stiffening, as determined by pulse wave velocity (Laurent et al., 2001; Mitchell et al., 2007; Mitchell, 2008; Palombo and Kozakova, 2016), which measures the rigidity of the arterial wall, as a whole (Armentano and Cymberknop, 2019), only few studies directly evaluated the impact of aging or CVD on the stiffness of the arterial endothelial layer. Huyn et al. (2011) provided an indirect evidence for age-related endothelial stiffening by demonstrating that denuded aortas (aortas in which endothelium is removed) of aged mice (20–25 months old) are significantly stiffer than denuded aortas of young mice (2–3 months old) and that this increase in sub-endothelial stiffness correlated with RhoA/ROCK-dependent disruption of the endothelial barrier. More recently, our studies demonstrated directly that endothelial monolayer of intact aortas is significantly stiffer in aortas isolated from aged mice (>10 months old), as compared to younger animals (3–6 months) (Le Master et al., 2018). Noteworthy, our recent study established that endothelial elastic modulus of aortas remains stable up until 6 months of age and then sharply increases in mice 10–12 months of age, representing “middle age” with further increase in the advanced aged animals (Le Master et al., 2022). Endothelial stiffening was also reported to accompany age-related macular degeneration in rhesus macaque (Cabrera et al., 2022). Thus, an increase in endothelial stiffness with age is well documented but the mechanisms responsible for these effects are still being investigated (Figure 4).

### 5.1 Endothelial stiffening as a secondary effect to arterial stiffening

The most well-studied, “canonical” mechanism of endothelial stiffening is a response to mechanical cues generated by a stiff substrate suggesting that endothelial stiffening with age is a secondary effect to the arterial stiffening (Tzima et al., 2001). Furthermore, the pathological consequences of age-related arterial stiffening are proposed to be attributed to the increased contractility/stiffening of the endothelium, which in turn is known to disrupt the junctional integrity of the endothelial monolayer (Huynh et al., 2011; Krishnan et al., 2011; Kohn et al., 2015b). This concept is based on two well-established phenomena, described above: 1) increased stiffness of the arterial wall with age, accompanied with endothelial stiffening, observed *in vivo* and 2) stiffening of endothelial cells grown on stiff substrates *in vitro*, which makes the connection between the two very logical but it is difficult to establish the causality between the two in aged vessels *in vivo*, as other factors may also play important roles, as will be discussed in the next sections.

### 5.2 Endothelial stiffening as a result of exposure to dyslipidemia

Our recent studies established that feeding mice high fat diet (HFD) leads to an increase in the elastic modulus of endothelium in intact mouse aortas, which mechanistically is mediated by the scavenger receptor CD36 (LeMaster et al., 2018), well-known to mediate the uptake of oxLDL and fatty acids (Silverstein and Febbraio, 2009). Surprisingly, and in contrast to existing paradigms, we discovered that global deletion of CD36 abrogates endothelial stiffening in mouse aortas of aged mice while having no effect on sub-endothelial stiffness suggesting that endothelial stiffening is driven by lipid uptake rather than substrate stiffness (Le Master et al., 2018). We also found that global deletion of caveolin-1 (Cav1), scaffolding protein required for the formation of caveolae, the primary mechanism of endocytosis of macromolecules into endothelial cells (Cohen et al., 2004; Jones and Minshall, 2020) and a known regulator of CD36 expression (Frank et al., 2004), results in the attenuation of endothelial stiffening in aortas of aged mice (Le Master et al., 2022). Thus, endothelial stiffening may not be secondary to arterial stiffening, as has been proposed in earlier studies, but may develop independently as a result of uptake and accumulation of oxidized lipids.

## 6 Relationship between endothelial stiffening and barrier function

Endothelial barrier integrity is modulated at the cell-cell interface by a variety of junctional factors, key among them proteins that stabilize anchors to a network of cortical actin bundles, the major element of the sub-membrane cytoskeleton (Garcia-Ponce et al., 2015; Karki and Birukov, 2019). The function of the barrier is to provide control of entry across the endothelial layer, offering protection against the entry of pathological factors while also providing control over extravasation of immune cells, such as leukocytes, into the vascular wall. Maintaining the integrity of the endothelial barrier relies on several variants of cell-cell junctions, the two main types of junctions are adherens junctions (AJs), and tight junctions (TJs) (Hartsock and Nelson, 2008; Tietz and Engelhardt, 2015; Adil et al., 2021). Adherens junctions consist of a complex of VE-cadherin, p120-catenin, and β-catenin linked to the cortical actin bundles located under the plasma membrane through association with N-WASP, ZO proteins, vinculin, among others. The key proteins of the tight junction are claudin and occludin and they are linked to cortical actin bundles *via* zona occludens (ZO) proteins. AJs are more numerous than TJs in the endothelial barrier interface.

The maintenance of the endothelial barrier and the stability of AJs/TJs critically depend on the organization and biomechanical features of the cytoskeleton, which might both reinforce the stability of the barrier or pull the junctions apart. In general, formation of cortical actin bundles, the sub-membrane cytoskeletal layer, connected to AJs/TJs reinforces the barrier whereas formation of contractile stress fibers, radial fibers that run across the cell and are normal to endothelial cell-cell interface, disrupt the barrier. Multiple studies have shown that activation of Rac1, a Rho-GTPase, that facilitate formation of cortical actin stabilizes and reinforces endothelial barrier, whereas activation of RhoA, a Rho -GTPase, that induce formation of contractile stress fibers *via* a phosphorylation cascade that phosphorylates Myosin II disrupts the barrier (Belvitch et al., 2018; Karki and Birukov, 2019; Wettschureck et al., 2019). Radial stress fibers pull on intercellular complexes of AJs and TJs *via* p120/β-catenin and ZO proteins respectively. In terms of endothelial stiffening, in theory, both formation of cortical actin and the contractile response may manifest themselves as increased cell stiffness/decreased deformability. Typically, however, endothelial stiffening is response to multiple environmental challenges was found to be mediated by RhoA-induced contractile response. As discussed in detail in the earlier sections of this review, activation of RhoA with subsequent phosphorylation of myosin and increased contractile response were found to underlie endothelial stiffening in response to hemodynamic forces, increased stiffness of the substrate and oxidized lipids. In one of our earlier studies, we evaluated the relative contributions of RhoA and Rac1 to oxLDL-induced stiffening and found that it entirely depends on RhoA and independent of Rac1 (Oh et al., 2016). Based on these observations, it is expected that endothelial stiffening is likely to be barrier disruptive.

Several studies have illustrated a direct association between endothelial stiffening and barrier disruption. Stroka et al. (2012) showed an increased contractility *via* MLCK phosphorylation, an enzyme that phosphorylates myosin II, to correlate directly with an increase in transvasation of neutrophils providing an early evidence for the role of contractile force generation in barrier disruption. A clear correlation between increased endothelial stiffness, as assessed by AFM, and an increase in transendothelial permeability assessed by electrical resistance (TEER) was shown in lung microvascular endothelial cells in response to thrombin *in vitro* (Suresh et al., 2019). This is consistent with the known effect of thrombin on RhoA activation and inducing stress fiber formation (Takeya et al., 2008; Giri et al., 2022). Interestingly, the authors also found that caspase-3, a protease primarily associated with programmed cell death (Asadi et al., 2022), partially prevents both the stiffening and the barrier disruption. Rokhzan et al. (2019) further characterized the impact of thrombin on endothelial contractile response in dermal microvascular endothelial cells by employing epifluorescence traction mapping to generate quantitative maps of force distribution within the endothelial cells, a method called Force Traction Microscopy (Wang and Lin, 2007) and showed that thrombin increased transendothelial permeability (TEER) in tandem with increases in contractile forces across the monolayer. The authors also showed, however, that contractile forces are not the only factor regulating the integrity of the barrier, as they were able to strengthen the barrier by treating the cells with angiopoietin-1, which increases VE-cadherin expression but has no effect on the contractile response.

The association between endothelial stiffening and barrier disruption was observed in a variety of clinical conditions, including hypertension and inflammation. Specifically, pressure-induced endothelial stiffening was accompanied by barrier disruption, as was assessed through two methods: TEER and neutrophil extravasation. The barrier disruption was observed in response to chronic high pressure, as manifested by an increase in TEER permeability, increased neutrophil extravasation, and discontinuous VE-cadherin. Similarly, induction of long-term (7–10 days) hyperglycemia *in vitro* results in the generation of radial stress fibers and cortical stiffening in endothelial cells, accompanied with a gap generations between the cells, as visualized by imaging of fluorescent actin, indicative of barrier disruption (Targosz-Korecka et al., 2013). Furthermore, after cells were returned to normoglycemic conditions for several days, both endothelial stiffness and barrier integrity improved in parallel even though some stiffening and gaps persisted. Endothelial stiffening was also observed in response to lipopolysaccharide or TNFα inflammatory signals with stiff stress fibers identified both by staining and by AFM mapping and 2D rendering (Okamoto et al., 2020). In this study, the integrity of adherens junctions was not evaluated but interestingly they showed that endothelial stiffening is accompanied with decreased functionality of the gap junctions, inter-cellular channels that allow passage of intracellular small molecules between adjacent cells suggesting that endothelial stiffening affects cell-cell communication/interaction *via* several mechanisms.

**FIGURE 5:**
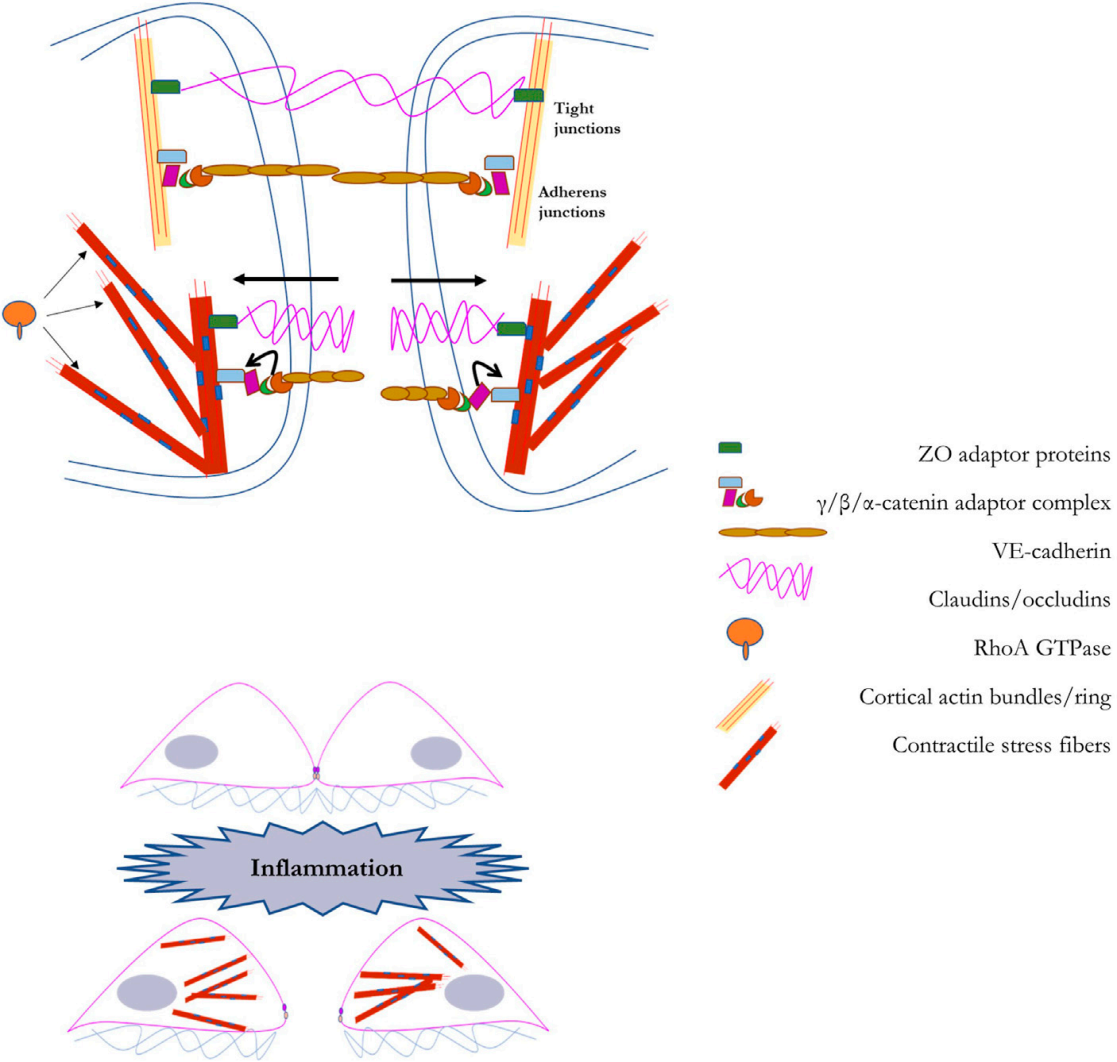
A schematic representation of the regulation of the endothelial barrier by the mechanical properties of endothelial cells: In the top panel we illustrate the barrier that couples adjacent endothelial cells. The endothelial barrier between adjacent cells provides a method for the controlled passage of molecules into the vascular wall. This barrier is primarily composed of tight junctions (composed of occludens and claudins) and adherens junctions (composed of VE-cadherin and catenins). The endothelial barrier maintains its integrity with scaffolding support from cortical actin fibers. In the bottom panel we illustrate the process through which barrier disruption is induced, such as during inflammation. *Via* RhoA-dependent generation of F-actin stress fiber networks, barrier degeneration is accomplished through force-based uncoupling of junctional proteins, leading to their sequestration, and facilitating gap formation between adjacent cells. A detailed explanation into the role of RhoA-dependent F-actin stress fiber networks in barrier degeneration is provided in Section 6 of the manuscript.

The mechanism behind endothelial stiffening/contractile-driven degeneration of the barrier is still not fully understood and continues to be studied. Several lines of evidence suggest contractile actin stress fibers apply tension on the elements of the adherens junctions resulting in the destabilization of the multiprotein complex (Figure 5). An early study of Birukova et al. (2012) demonstrated that a contractile response correlates with the phosphorylation of VE-cadherin, and its subsequent internalization away from the barrier interface, suggesting that mechanical forces may cause the loss of membrane expression of the major junctional proteins. A more detailed mechanism emerged from more recent studies that evaluated the effects of tension on the conformation state of VE-cadherin complex. Kugelmann et al., 2018 demonstrated that disruption of the endothelial barrier induced by thrombin or histamine is accompanied with the exposure of a tension-sensitive epitope of α-catenin, a linker protein that connects VE-cadherin complex to the actin cytoskeleton (Meng and Takeichi, 2009). The role of α-catenin in force-induced reorganization of the junctions was discovered in an earlier study by Yonemura et al., 2010 who showed that tension force applied by actin fibers to the C-terminus of α-catenin results in the change of α-catenin conformation or unwinding to expose a vinculin-binding region. Yonemura et al. (2010) also generated a tension-sensitive α-catenin antibody that binds to the epitope of α-catenin exposed in response to tension. Using this tension-sensitive α-catenin antibody, Kugelmann et al. (2018) showed that tension on α-catenin develops simultaneously with endothelial barrier disruption, as estimated by both imaging VE-cadherin organization/jaggedness and TEER. Inhibition of the ROCK activity during stimulation with thrombin/histamine abrogated tensional force generation on α_18_-catenin, demonstrating that RhoA/ROCK signaling is essential to eliciting direct tensional force on VE-cadherin complexes. They did not observe any significant change in vinculin distribution, however, suggesting the role of α-catenin tension may have a more general effect on the reorganization of adherens junctions beyond the association with vinculin. Interestingly, Kugelmann et al. (2018) found that as tensional force on α_18_-catenin increases, there is increased translocation of RhoA, as assessed by FRET, to the cell border, which might further contribute to increased tension. In addition, they found that chelation of Ca^2+^ during stimulation with thrombin or histamine abrogate tensional force generation on α_18_-catenin and histamine-induced RhoA activation, demonstrating that Ca^2+^ mediated RhoA/ROCK signaling is essential to eliciting direct tensional force on VE-cadherin complexes of the endothelial barrier.

Another direct link between actomyosin contractile response and disruption of adherens junctions was found by Arif et al., 2021 who demonstrated that tension generated by actin fibers result in unmasking a VE-cadherin phosphorylation site, tyrosine 731, that is masked by catenin under resting conditions. This unmasking is also accompanied by tension developing within VE-cadherin itself, as indicated by a tension FRET sensor. Most importantly, unmaking of tyrosine 731 makes it accessible for SHP2 phosphatase, which results in its dephosphorylation and subsequent VE-cadherin internalization. Activation of SHP2 is induced by leukocyte binding to endothelial cells through PECAM1, a known endothelial mechanosensory. It is thus, possible, that tension plays a dual role in the disruption of the barrier and diapedesis of leukocytes across the endothelial layer: unmasking VE-cadherin phosphorylation site by actin-mediated contractile forces and activation of SHP2 by tension-induced activation of PECAM1.

## 7 Concluding remarks and future directions

Multiple studies demonstrated that endothelial cells stiffen in response to mechanical and soluble factors. The most well-studied and the dominant mechanism of endothelial stiffening is RhoA/Myosin-dependent formation of actin stress fibers and contractile response. There is also accumulating amount of evidence showing that cortical actin and intermediate filaments may contribute to endothelial stiffening but the relative contributions of these mechanisms to endothelial stiffening in response to physiological or pathological stimuli is still not well understood.

This review also presents our current understanding on the contribution of mechanical signals towards endothelial stiffening. Consistent with the dominant role of RhoA-induced stress fiber formation, this mechanism is induced by the mechanical signals, which include fluid shear stress, stretch and increase in matrix stiffness. It is important to note, however, that the same signals also induce Rac1 activation, which has an inverse relationship with RhoA, and further evidence is required to understand the biphasic role of RhoA/Rac1 activation and the mechanism driving cell remodeling of the biophysical properties of extracellular matrix. Another important outstanding question is what is the differential response of the anti-inflammatory unidirectional and pro-inflammatory non-unidirectional flow on endothelial stiffness. We also highlight the role of oxidized lipids in endothelial stiffening, which is relatively less studied and recognized. In this context, it is interesting to note that non-unidirectional flow was shown to increase endothelial stiffness in oxLDL-dependent way, by enhancing oxLDL uptake and thus, oxLDL-induced endothelial stiffening. Furthermore, there is clear evidence that the uptake of oxidized lipids plays a key role in endothelial stiffening induced by high fat diet and by aging. The relative contributions and the crosstalk of mechanical and soluble lipid signals to endothelial stiffening under different pathological conditions need further study. Lastly, we focused on reviewing the role of endothelial stiffness in maintenance of the barrier integrity. While extensive research showed that RhoA-induced contractile stress fiber formation results in the disruption of the endothelial barrier, only few studies present direct evidence into the role of endothelial stiffening in barrier disruption induced by hyperglycemia, inflammation, and high pressure. In addition, we present emerging evidence into the mechanism of RhoA-induced stress fiber formation in barrier disruption, a mechanism that shows that tensional force strength is a factor in eliciting the degeneration of adherens junction protein complexes. The role of endothelial stiffening into eliciting barrier disruption, specially that induced by aging or vascular disease, needs further study.
